# Crassulacean acid metabolism-cycling in *Euphorbia milii*

**DOI:** 10.1093/aobpla/plt014

**Published:** 2013-03-04

**Authors:** Ana Herrera

**Affiliations:** Instituto de Biología Experimental, Facultad de Ciencias, Universidad Central de Venezuela, Caracas, Miranda 1020, Venezuela

**Keywords:** CAM-cycling, citrate, transpiration, water saving, water-use efficiency

## Abstract

Crassulacean acid metabolism (CAM) occurs in many Euphorbiaceae, particularly *Euphorbia*, a genus with C_3_ and C_4_ species as well. With the aim of contributing to our knowledge of the evolution of CAM in this genus, this study examined the possible occurrence of CAM in *Euphorbia milii*, a species with leaf succulence and drought tolerance suggestive of this carbon fixation pathway. Leaf anatomy consisted of a palisade parenchyma, a spongy parenchyma and a bundle sheath with chloroplasts, which indicates the possible functioning of C_2_ photosynthesis. No evidence of nocturnal CO_2_ fixation was found in plants of *E. milii* either watered or under drought; watered plants had a low nocturnal respiration rate (*R*). After 12 days without watering, the photosynthetic rate (*P*_N_) decreased 85 % and nocturnal *R* was nearly zero. Nocturnal H^+^ accumulation (ΔH^+^) in watered plants was 18 ± 2 (corresponding to malate) and 18 ± 4 (citrate) μmol H^+^ (g fresh mass)^−1^. Respiratory CO_2_ recycling through acid synthesis contributed to a night-time water saving of 2 and 86 % in watered plants and plants under drought, respectively. Carbon isotopic composition (δ^13^C) was −25.2 ± 0.7 ‰ in leaves and −24.7 ± 0.1 ‰ in stems. Evidence was found for the operation of weak CAM in *E. milii*, with statistically significant ΔH^+^, no nocturnal CO_2_ uptake and values of δ^13^C intermediate between C_3_ and constitutive CAM plants; ΔH^+^ was apparently attributable to both malate and citrate. The results suggest that daily malate accumulation results from recycling of part of the nocturnal respiratory CO_2_, which helps explain the occurrence of an intermediate value of leaf δ^13^C. *Euphorbia milii* can be considered as a CAM-cycling species. The significance of the operation of CAM-cycling in *E. milii* lies in water conservation, rather than carbon acquisition. The possible occurrence of C_2_ photosynthesis merits research.

## Abstract (precis)

Photosynthetic characteristics of *Euphorbia milii* are reported for the first time. The occurrence of CAM-cycling is shown to serve as a mechanism of water conservation. This is a detailed and novel report of such CAM mode in the genus, which is abundant in constitutive CAM and C_4_ species. Anatomical evidence for the possible operation of a CO_2_-concentrating mechanism around the vascular bundle, the C_2_ route, is provided. Findings are important for our understanding of evolution of CAM in the genus.

## Introduction

Crassulacean acid metabolism (CAM) is of frequent occurrence among the Euphorbiaceae and has appeared polyphyletically several times in the family, particularly in the genus *Euphorbia*. In this genus, C_4_ species seem to be rare, whereas they are abundant in the genus *Chamaesyse* (Webster *et al*. 1975). In *Euphorbia*, CAM has been reported in 21 species, and values of δ^13^C suggest its presence in 44 species (Table 1). Several of these species belong to three different clades within the genus (cladograms in Zimmermann *et al*. 2010). Twenty-four species can be considered constitutive CAM on the basis of having values of δ^13^C higher than −17 ‰, a criterion established by Mooney *et al*. (1977). In the remaining species, values of δ^13^C average −24.7 ‰. A value as low as −28.9 ‰ found in *E. aphylla* falls into the lower mode of the bimodal frequency distribution of δ^13^C in CAM plants, designated as low-level (weak) CAM (Winter and Holtum 2002; Silvera *et al*. 2005).

**Table 1:** Carbon isotopic composition of species of *Euphorbia* with high to intermediate values of δ^13^C and CAM mode assigned by authors on the basis of leaf gas exchange, acid accumulation, δ^13^C and enzyme activity.

Species	δ^13^C (‰)	Mode	Reference
*angusta*	−24.9	NA	Webster *et al*. (1975)
*antiquorum*	−14.2	NA	Batanouny *et al*. (1991)
*aphylla*	ND	Facultative	Mies *et al*. (1996)
*avasmontana*	−15.1	Constitutive	Mooney *et al.* (1977)
*bothae*	−14.6	Constitutive	Mooney *et al.* (1977)
*bubalina*	−13.2	NA	Webster *et al*. (1975)
*burmannii*	−18.3	NA	Mooney *et al.* (1977)
*caducifolia*	ND	Constitutive	Webster *et al*. (1975), Sayed (2001)
*caput-medusae*	−13.3	Constitutive	Mooney *et al.* (1977)
*cyathophora*	−25.8	NA	Mies *et al*. (1996)
*didieroides*	−26.6	NA	Webster *et al*. (1975)
*didieroides*	−24.3	NA	Winter (1979)
*dregeana*	ND	Constitutive	Sayed (2001)
*drupifera*	−14.1	NA	Webster *et al*. (1975)
*gariepina*	−14.6	Constitutive	Mooney *et al.* (1977)
*genoudiana*	−22.7	NA	Winter (1979)
*gorgonis*	−12.9	Constitutive	Mooney *et al.* (1977)
*gragaria*	−11.6	Constitutive	Mooney *et al.* (1977)
*grandidens*	ND	Constitutive	Webster *et al*. (1975), Sayed (2001)
*inermis*	−13.4	Constitutive	Mooney *et al.* (1977)
*ingezalahiana*	−23.6	NA	Winter (1979)
*inocua*	−28.1	NA	Webster *et al*. (1975)
*leucodendron*	−13.2	NA	Winter 1979
*macropodoides*	−28.3	NA	Webster *et al*. (1975)
*macropus*	−28.9	NA	Webster *et al*. (1975)
*mauritanica*	−16.0	Constitutive	Mooney *et al.* (1977)
***milii***	**ND**	**Non-CAM**	**Webster *et al*. (1975)**
***milii***		**CAM**	**McWilliams (1970)**
*nesemannii*	−11.6	Constitutive	Mooney *et al.* (1977)
*nivulia*	−15.7	NA	Webster *et al*. (1975)
*nubica*	−14.5	NA	Batanouny *et al*. (1991)
*pentagona*	−14.9	Constitutive	Mooney *et al.* (1977)
*peperomioides*	−25.6	NA	Webster *et al*. (1975)
*plagiantha*	−13.2	NA	Winter (1979)
*polygona*	−10.7	Constitutive	Mooney *et al.* (1977)
*pulcherrima*	−25.5	NA	Mies *et al*. (1996)
*squarrosa*	−12.5	Constitutive	Mooney *et al.* (1977)
*stenoclada*	−12.6	NA	Winter (1979)
*submamillaris*	ND	Constitutive	Webster *et al*. (1975), Sayed (2001)
*tetragona*	−14.7	Constitutive	Mooney *et al.* (1977)
*thi*	−13.2	NA	Batanouny *et al*. (1991)
*tirucalli*	−15.3	Constitutive	Mies *et al*. (1996)
*triangularis*	−13.6	Constitutive	Mooney *et al.* (1977)
*trigona*	−19.4	NA	Webster *et al*. (1975)
*xylophylloides*	ND	Constitutive	Webster *et al*. (1975), Sayed (2001)

NA, no mode assigned; ND, not determined.

Since values of δ^13^C alone are not sufficient to distinguish between C_3_ species and plants that obtain up to one-third of their carbon during the night, which include weak CAM plants (Winter and Holtum 2002), measurements of physiological and biochemical variables are necessary. In order to demonstrate the operation of CAM, routine determinations include, among others, ΔH^+^, δ^13^C and nocturnal CO_2_ fixation. Griffiths *et al*. (2007) devised an ingenious method of ascertaining the occurrence of nocturnal CO_2_ fixation by examining the response of the night-time CO_2_ exchange rate to intercellular CO_2_ concentration (*C*_i_).

Intermediate values of δ^13^C can also suggest the occurrence of C_3_ metabolism with high water-use efficiency, as the data of Farquhar and Richards (1984) on wheat indicate, or of C_2_ photosynthesis, as in the case of *Euphorbia acuta*. In wheat and maize, C_2_ photosynthesis is responsible for an increase of 8–11 % in photosynthetic rate through re-assimilation of photorespired CO_2_ (Busch *et al*. 2013).

Plants of *Euphorbia milii* subgenus *Euphorbia*, Section *Goniostema*, common name crown of thorns, originally from Madagascar, are cultivated worldwide for their ornamental value. Plants are perennial armed shrubs as tall as 1 m, with fleshy stem and branches, and partly succulent leaves. According to observations by Mooney *et al*. (1977), CAM is present in the weak mode in leafy species of the genus. The medicinal and molluscicidal properties of the latex in *E. milii* have been extensively investigated (e.g. Mwine and Van Damme 2011); in contrast, literature on the physiology of the species is practically non-existent.

In spite of the succulence of its leaves and the various reports of CAM in the genus, *E. milii* has been reported as non-CAM (Webster *et al*. 1975). Nevertheless, recalculation of the data of McWilliams (1970) gives a ΔH^+^ of 100 μmol (g fresh mass)^−1^ and a dark CO_2_ fixation rate of 0.1 μmol m^−2^ s^−1^, suggesting that CAM in *E. milii* operates in the cycling mode, i.e. nocturnal H^+^ accumulation and daytime but nearly no night-time CO_2_ fixation (for the definition of CAM modes, see Cushman 2001).

With the aim of contributing to our knowledge of the evolution of CAM in *Euphorbia*, this study re-examined the possible occurrence of CAM in *E. milii* through daily leaf gas exchange, including *P*_N_/*C*_i_ and *R*/*C*_i_ curves (where *P*_N_ is the photosynthetic rate and *R* is the respiration rate), measurements of dawn and dusk H^+^ content, and determinations of δ^13^C.

## Methods

### Plant material and cultivation

Plants of *E. milii* were propagated from one plant purchased at a nursery by inserting cuttings into the soil of 2-L pots filled with silty clay loam (Viveros Exotica Raphia, S.R.L., Caracas); plants were fertilized monthly with N : P : K 15 : 15 : 15 and grown in the garden for 1 year before the beginning of experiments. Plants, ∼50 cm tall, were maintained in the greenhouse under natural light, fully watered every other day and fertilized weekly. Day length was 12 h (06:00–18:00 h), mean maximum daily photosynthetic photon flux density (PPFD) between 09:00 and 14:30 h 507 ± 22 μmol m^−2^ s^−1^, mean air temperature 32 ± 5/18.4 ± 0.5 °C (day/night) and relative humidity 60 ± 10 %. Water deficit was imposed by withholding watering.

### Anatomy

Free-hand cross-sections of stems (average thickness 5 mm) and leaves were observed under the microscope at ×40 (stem) and ×400 (leaf). Leaf sections were stained with toluidine blue.

### Succulence

Leaf and stem water content was determined as the difference between the fresh mass (FM) and the mass after drying for 72 h at 60 °C [dry mass (DM)], divided by the area in the case of leaves (FM/A) and by DM in the case of stems. Leaf thickness was measured with precision calipers. Chlorophyll (Chl) content was determined after Bruinsma (1963) in 80 % cold acetone extracts of leaf or stem sections collected at 18:00 h. The mesophyll succulence index was calculated as *S*_m_ = g water (mg Chl)^−1^ after Kluge and Ting (1978).

### Stable carbon isotope composition

The δ^13^C was determined with a precision of 0.15 ‰ using a ThermoFinnigan DeltaPlusXL Isotope Ratio Mass Spectrometer (San Jose, CA, USA) and PDB as the standard.

### Nocturnal H^+^ accumulation

Whole leaves were weighed fresh and set to boil in 50 mL distilled water for 10 min in a microwave oven at maximum power; samples were sieved through a plastic colander, leaf segments and the colander were rinsed, and the solution was made up to 100 mL. Samples were titrated to pH 7.0 for the estimation of H^+^ corresponding to malate according to Nobel (1988), and to pH 8.4 for citrate. Since Franco *et al*. (1990) noted that there was a strong linear relationship between concentrations of malate and citrate determined enzymatically and by titration, in the absence of an enzymatic method for the determination, titration is an adequate alternative. Latex was collected from cut stems and leaves, suspended in distilled water and titrated likewise. The ΔH^+^ was calculated as the difference between dawn and dusk H^+^ contents.

### Leaf gas exchange

The *P*_N_, *R*, stomatal conductance (*g*_s_) and transpiration rate (*E*) were measured in the laboratory with a CIRAS 2 IRGA connected to a PLC(B) assimilation chamber (PP Systems, Amesbury, MA, USA) at an incoming CO_2_ concentration (*C*_a_) of 380 μmol mol^−1^, a chamber temperature tracking ambient (24 ± 1 °C) and an incident PPFD of 200 (the first morning hours) or 1000 μmol m^−2^ s^−1^ (the rest of the daytime). Records were automatically taken every 30 min. Response curves were done in six different leaves of *P*_N_ and *C*_i_ between 10:00 and 11:00 h, and of nocturnal CO_2_ exchange to *C*_i_ between 20:00 and 05:00 h.

### Statistics

Values are mean ± SE (*n* = 6). Statistical significance was assessed where indicated through one- or two-way analysis of variance (ANOVA) (*P* < 0.05) with the Statistica package.

## Results

**Figure 1.**
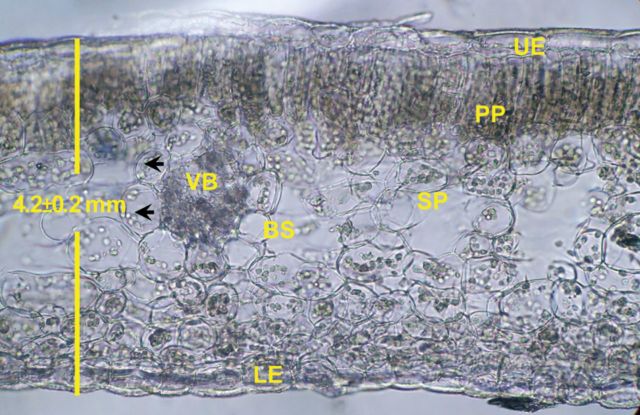
Cross-sections of the leaf of *E. milii*. UE, upper epidermis; PP, palisade parenchyma; VB, vascular bundle; BS, bundle sheath; SP, spongy parenchyma; LE, lower epidermis. Arrowheads point at chloroplasts.

Leaf cross-sections showed a dorsiventral anatomy, with a compact palisade parenchyma containing many chloroplasts and a spongy parenchyma with large vacuoles and fewer chloroplasts; the spongy parenchyma constituted 40 % of the whole-leaf thickness (Fig. 1). A bundle sheath with large chloroplasts located centrifugally was observed. Cross-sections of the fleshy stems (not shown) have a thick green cortex and colourless pith; the cortex was 54 % of the stem thickness on average.

In watered plants, *S*_m_ in both leaves and stem green cortex was 2.7 ± 0.2 g water (mg Chl)^−1^; δ^13^C was −25.2 ± 0.7 ‰ in leaves and −24.7 ± 0.1 ‰ in stems. In the stem green cortex of watered plants, malate- and citrate-H^+^ content was 48 ± 9 and 29 ± 5 μmol H^+^ (g FM)^−1^, respectively, without daily oscillation. Water suspensions of latex showed no acid content.

**Figure 2.**
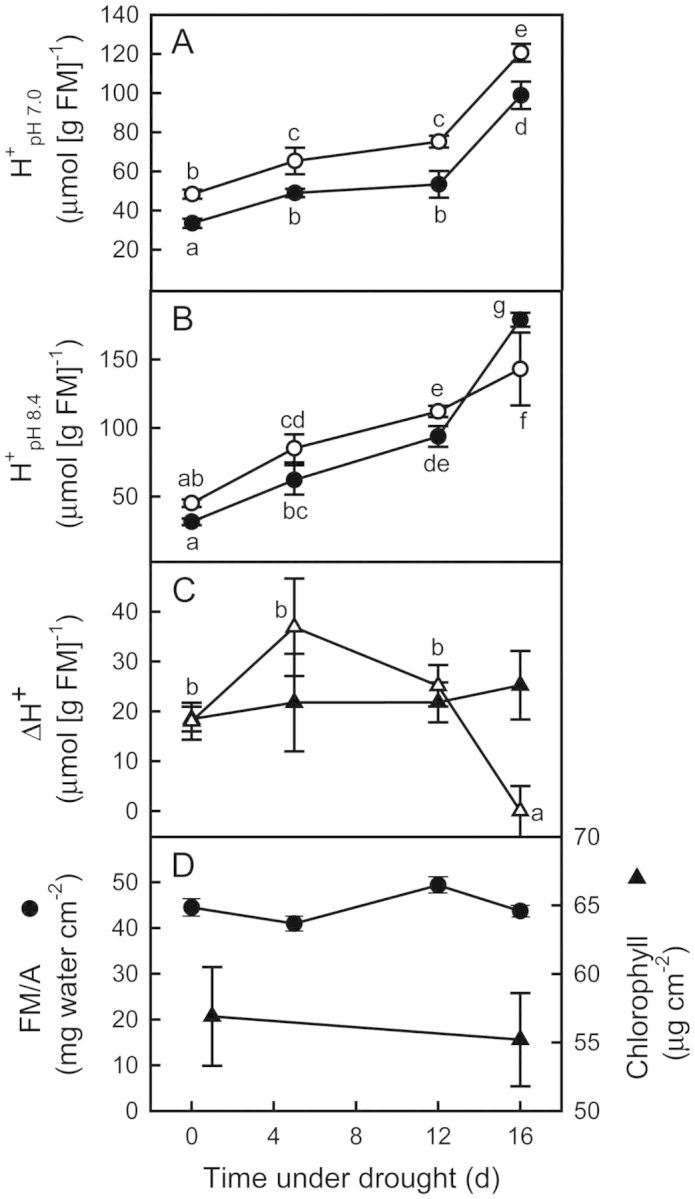
Time course of changes with drought in leaves of *E. milii* in (A) H^+^ content titrated to pH 7.0 (empty circles, dawn; filled circles, dusk); (B) H^+^ content titrated to pH 8.4 (empty circles, dawn; filled circles, dusk); (C) nocturnal H^+^ accumulation (empty triangles, pH 8.4; filled triangles, pH 7.0), and (D) dawn leaf FM per area (circles) and chlorophyll content (triangles). Values are mean ± SE (*n* = 12). Different letters indicate significant differences at *P* < 0.05 after a two-way ANOVA (time under drought × hour of day for each pH in A and B) and a one-way ANOVA (time under drought for each pH in C).

Leaves had significant amounts of malate- and citrate-H^+^ at dawn and dusk, contents increasing with time under drought (Fig. 2A and B). A significant accumulation of malate-H^+^ took place in watered plants, which remained constant up to 16 days of drought (*P* < 0.05). A similar trend in dawn and dusk H^+^ content and ΔH^+^ for citrate-H^+^ was found, except that after 16 days of drought ΔH^+^ became zero. Mean ΔH^+^ was 18 ± 2 (malate) and 18 ± 4 (citrate) μmol (g FM)^−1^. Changes in either morning and evening H^+^ contents or ΔH^+^ bore no relationship to changes in FM/A, which remained relatively constant for the duration of the experiment, as did Chl content (Fig. 2). The ratio Chl a/b remained unchanged at 3.3 ± 0.2. Stem water content was 11.2 ± 0.5 g water (g DM)^−1^, twice as high as in leaves, and did not vary with time under drought (*P* = 0.86).

**Figure 3.**
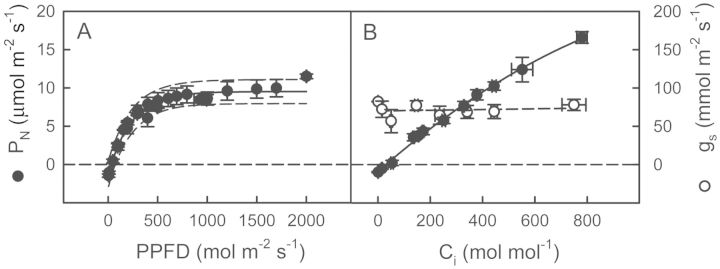
Response curve of the leaf photosynthetic rate to (A) photosynthetic photon flux density and (B) leaf intercellular CO_2_ concentration in watered plants of *E. milii*. Values are mean ± SE (*n* = 6). Filled circles, *P*_N_; empty symbols, *g*_s_. Measurements were made at a CO_2_ concentration of 380 μmol mol^−1^ in (A) and a PPFD of 1000 μmol m^−2^ s^−1^ in (B).

As shown in Fig. 3A, *P*_N_ of watered plants became saturated at 700 μmol m^−2^ s^−1^ PPFD; apparent quantum yield was 0.047 and light-compensation point 48 μmol m^−2^ s^−1^. The *P*_N_/*C*_i_ curves (Fig. 3B) show that *P*_N_ did not become saturated by *C*_i_, increasing 47 % with an increase in *C*_i_ to 800 μmol mol^−1^ (*C*_a_ = 1240 μmol mol^−1^). This lack of saturation could have been due to very low *g*_s_, which remained unchanged by *C*_i_. The CO_2_ compensation concentration was 32 μmol mol^−1^.

**Figure 4.**
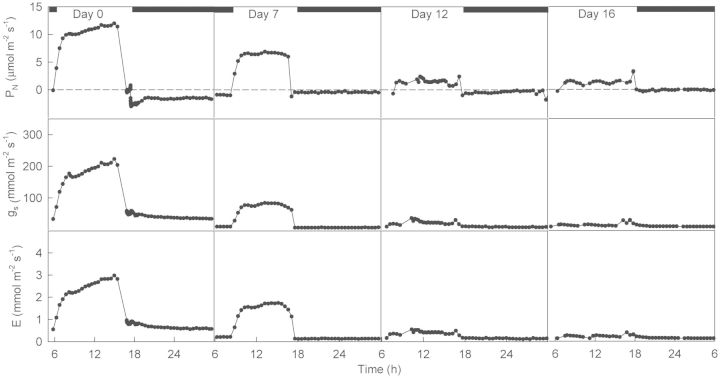
Daily course of the leaf photosynthetic rate, stomatal conductance and transpiration rate in plants of *E. milii* under drought for 0, 7, 12 and 16 days. Measurements were made at a CO_2_ concentration of 380 μmol mol^−1^, leaf temperature of 24.0 ± 1.0 °C and PPFD of 200 (06:00–10:00 h) and 1000 μmol m^−2^ s^−1^ (10:00–18:00 h). The dark bar on the abscissa indicates the length of the dark period.

Daily courses of leaf gas exchange done in plants progressively under drought showed a decrease in *P*_N_ of 85 % with drought; *R* became nearly zero after 12 and up to 16 days without watering (Fig. 4). Mean daytime water-use efficiency calculated from these courses of leaf gas exchange was relatively high, decreasing significantly with drought only 18 %, from 4.2 ± 0.1 to 3.5 ± 0.1 mmol mol^−1^ (*P* = 0.00).

Stem cross-sections of 1.5 cm^2^ average area from watered plants introduced in the assimilation chamber showed daytime CO_2_ assimilation at rates similar to those determined in leaves on a Chl basis (Table 2). Stem sections, as opposed to leaves, showed dark CO_2_ uptake.

**Table 2:** Photosynthetic and respiration rate of stem cross-sections inserted into the IRGA assimilation chamber.

Organ	PPFD (μmol m^−2^ s^−1^)	*P*_N_	
		(μmol m^−2^ s^−1^)	(μmol (g Chl)^−1^ s^−1^)
Leaf	1500	9.9 ± 1.2	15.1 ± 0.3
Stem	1500	10.0 ± 0.2	9.1 ± 1.9
Leaf	0	−1.5 ± 0.2	−2.3 ± 0.3
Stem	0	0.5 ± 0.2	4.7 ± 1.3

Values are mean ± SE (*n* = 6). Incident photosynthetic photon flux density is indicated.

**Figure 5.**
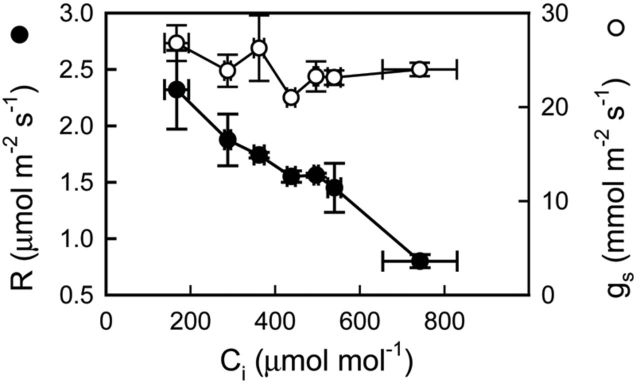
Response curves to leaf intercellular CO_2_ concentration of nocturnal respiration rate and stomatal conductance in watered plants of *E. milii*. Filled symbols, *R*; empty symbols, *g*_s_. Values are mean ± SE (*n* = 6).

A significant decrease of 65 % in *R* with *C*_i_ was observed without significant changes in *g*_s_ (Fig. 5).

**Figure 6.**
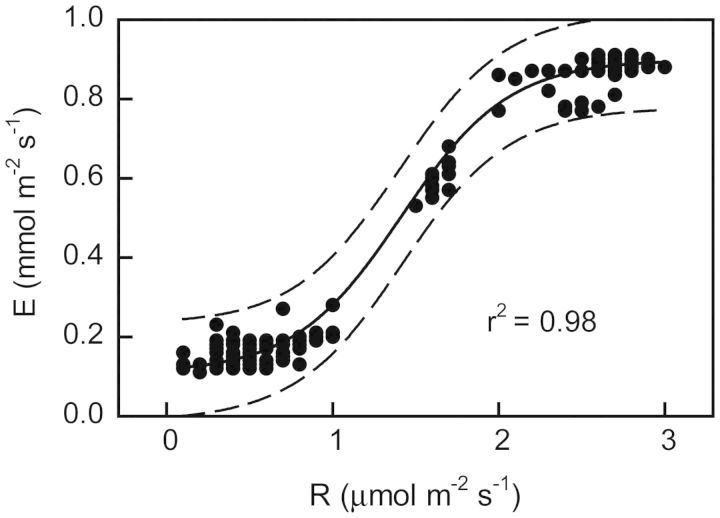
Change in nocturnal leaf transpiration rate of plants of *E. milii* watered and under different degrees of drought as a function of nocturnal respiration rate. Values are data points. The regression line (solid), 95 % confidence intervals (broken lines) and determination coefficient (*P* < 0.05) are shown.

The regression of *E* vs. *R* is shown in Fig. 6. Assuming that the accumulated acids were the products of the recycling of respiratory CO_2_, the absolute recycling, i.e. the amount of CO_2_ contained in acids, was calculated. Together with the *E* vs. *R* regression, it was found that recycling recovered 10 and 37 % of nocturnal CO_2_ loss in watered plants and after 12 days of drought, respectively, and helped in saving water during the night by 15 % in watered plants and 2 % in plants under drought. Daytime water saving, calculated as the ratio of the absolute amount of CO_2_ in acid equivalents to integrated *P*_N_ (after Fetene and Lüttge 1991), was 2 and 86 % in watered plants and plants under drought, respectively.

## Discussion

Evidence was found for the operation of weak CAM in *E. milii*, with statistically significant ΔH^+^ in watered plants and plants under drought, low δ^13^C and no nocturnal CO_2_ uptake; ΔH^+^ was apparently attributable to both malate and citrate. Results suggest that daily malate and citrate accumulation results from recycling of part (watered plants) or all (plants under drought) of the nocturnal respiratory CO_2_. Recycling of CO_2_ through malate synthesis, together with the absence of nocturnal CO_2_ uptake, helps explain the occurrence of values of leaf δ^13^C intermediate between C_3_ and constitutive CAM plants.

Values of ΔH^+^ determined at pH 7.0 were low compared with CAM species such as *Kalanchoe tubiflora* and *Clusia minor*, comparable with the cycling species *T. parviflorum* and *T. mengessi* and not as low as in *Talinum teretifolium* or *Zamioculcas zamiifolia* (Table 3).

**Table 3:** Values of nocturnal acid accumulation and carbon isotopic composition reported for CAM species.

CAM mode	Species	ΔH^+^	δ^13^C	Reference
		(μmol (g FM)^−1^)	(‰)	
Facultative	*Clusia minor*	1400	−24.6	Borland *et al*. (1992, 1994)
Constitutive	*Kalanchoe daigremontiana*	152	−16.7	Holtum *et al*. (1983)
Cycling	*Talinum calycinum*	39	−25.2	Martin and Zee (1983)
Cycling	*Sedum nuttalianum*	37	−27.2	Gravatt and Martin (1992)
Cycling	*Talinum calcaricum*	29	−26.0	Harris and Martin (1991)
Cycling	*Sedum telephioides*	23	−26.2	Gravatt and Martin (1992)
**Cycling**	***Euphorbia milii***	**18**	**−25.2**	**This study**
Cycling	*Talinum teretifolium*	14	−25.4	Harris and Martin (1991)
Cycling	*Talinum parviflorum*	11	−25.8	Harris and Martin (1991)
Cycling	*Talinum mengessi*	6	−24.3	Harris and Martin (1991)
Facultative	*Zamioculcas zamiifolia*	5	ND	Holtum *et al*. (2007)

Some values were re-calculated from the data in references. ND, not determined.

A value of *S*_m_ higher than 1 g water (mg Chl)^−1^ was also suggestive of CAM, as proposed by Kluge and Ting (1978). The succulent nature of leaves was corroborated by the microscopic observation of cross-sections, in which cells with a large volume and few chloroplasts are present, as in many leaf-succulent CAM plants (Kluge and Ting 1978). In facultative CAM species, values of *S*_m_ are intermediate (Table 4) but, given that low as well as high values of *S*_m_ can be found in constitutive CAM species (Table 4), it becomes apparent that for a leaf to perform full CAM a large proportion of vacuole volume to chloroplasts is not required. The lack of significant differences in FM/A between five strong CAM and three weak CAM species (Nelson and Sage 2008) lends support to this hypothesis.

**Table 4:** Values of mesophyll succulence index reported for constitutive, facultative- and cycling-CAM species.

Species	*S*_m_ g water (mg Chl)^−1^	CAM mode	Reference
*Kalanchoe daigremontiana*	1.3	Constitutive	Kluge and Ting (1978)
***Euphorbia milii***	**2.7**	**Cycling**	**This study**
*Talinum paniculatum*	3.4	Facultative	Güerere *et al*. (1996)
*Talinum triangulare*	6.0	Facultative	Herrera *et al*. (1991)
*Puya floccosa*	6.2	Facultative	Herrera *et al*. (2010)
*Sedum morganianum*	13.0	Constitutive	Kluge and Ting (1978)

The presence in *E. milii* of bundle sheath cells with chloroplasts is indicative of the possible operation of C_2_ photosynthesis, as reported in *E. acuta*. This species shares with *E. milii* low, C_3_-like values of δ^13^C (−25.5 ‰ according to Webster *et al*. 1975, and −28.5 ‰ according to Sage *et al*. 2011) and an intermediate value of CO_2_ compensation concentration (32 mmol mol^−1^; Sage *et al*. 2011). The occurrence of CAM, Kranz anatomy and C_4_ photosynthesis in the same leaf has been reported in Portulacaceae (Guralnick and Jackson 2001), but to date no report on CAM together with C_2_ photosynthesis has been published. Given that the C_2_ route of carbon fixation has been proposed as an intermediate evolutionary step from C_3_ to C_4_ (Sage *et al*. 2011), investigating the functioning of C_2_ photosynthesis in *E. milii* would bring interesting viewpoints on the evolution of CAM and C_4_ plants, particularly in *Euphorbia*.

A significant oscillation in H^+^ content corresponding to citrate was found, equivalent to 12 μmol citrate (g FM)^−1^ after 12 days of drought, comparable with the lower end, ∼22 μmol (g FM)^−1^, of the range in species of *Clusia*, a genus abundant in CAM species performing different modes (Lüttge 2007). The role of citrate accumulation in carbon or water balance during CAM remains unclear (Lüttge 2007); citrate does not provide net CO_2_ gain, as does malate, but should prove more effective than malate in increasing *C*_i_ during the day because its breakdown produces three molecules of CO_2_ as opposed to one in the case of malate (Lüttge 2007). Increased *C*_i_ during the day is a photoprotective mechanism well recognized in CAM plants, as shown in plants of the facultative CAM species *Talinum triangulare* under drought (Pieters *et al*. 2003).

Photosynthetic characteristics in *E. milii* were consistent with those of a sun plant: high apparent quantum yield, saturating PPFD and Chl a/b ratio (Pearcy and Franceschi 1986).

The absence of net dark CO_2_ fixation was consistent with the proportion of dark CO_2_ uptake calculated using the regression equations of the proportion of CO_2_ fixed during the night against δ^13^C found by Winter and Holtum (2002) and Pierce *et al*. (2002). In many facultative CAM species, δ^13^C tends towards low values. In a review of 23 facultative CAM species (Herrera 2009), the mean, maximum and minimum δ^13^C were −23.9, −14.0 and −30.0 ‰, respectively, indicating that the variability in δ^13^C values may lead researchers to classify a species as a C_3_, facultative or constitutive CAM plant. In *Euphorbia aphylla*, δ^13^C ranged from −27.1 ‰ for the youngest cladode in the dry season during summer to −24.5 ‰ for the oldest cladode in the rainy season during winter (Mies *et al*. 1996), reflecting the effects on δ^13^C of day/night temperatures, water availability and developmental stage.

Values of δ^13^C higher in stems than in leaves suggest the occurrence of nocturnal CO_2_ fixation, although this could not be demonstrated in intact plants. The occurrence of an assimilation rate in the dark amounting to a third of PPFD-saturated leaf *P*_N_ strongly suggested that stems are capable of nocturnal CO_2_ fixation. Stem internal CO_2_ re-fixation in young twigs and branches possessing a green cortex may compensate for 60–90 % of the potential respiratory carbon loss (Pfanz *et al*. 2002). If stem recycling in *E. milii* occurred through phosphoenolpyruvate carboxylase (PEPC) activity, that would explain the apparent ^13^C enrichment. There are two alternative explanations for higher δ^13^C in the stems of *E. milii*. One explanation is that this variable was determined in sections comprising all tissues, green as well as non-autotrophic; non-autotrophic organs of C_3_ plants, such as stems, have been found to be enriched in δ^13^C by ∼1–3 ‰ relative to leaves (Cernusak *et al*. 2009). Another, simpler, explanation is that barriers to CO_2_ diffusion into the stem are larger than into leaves, hindering entrance of the heavier ^13^CO_2_. Actual nocturnal CO_2_ fixation by stems of *E. milii* remains to be determined accurately by methods such as carbon labelling, involving all stem tissues.

The response of leaf dark respiration to *C*_i_ suggests the operation of a carboxylation system, most likely PEPC, which makes recycling of respiratory CO_2_ possible. In the constitutive CAM plant *Kalanchoe daigremontiana*, a *P*_N_/*C*_i_ curve during phase I of the CAM cycle showed a pronounced increase at low *C*_i_ and saturation at a *C*_i_ of ∼250 μmol mol^−1^ (Griffiths *et al*. 2007). Our results show that the response of dark respiration to *C*_i_ was not an artefact caused by changes in *g*_s_, because *g*_s_ remained constant in spite of increasing *C*_i_.

Water saving through respiratory CO_2_ recycling was significant, as in the case of *T. paniculatum*, a facultative CAM species, in which the amount of water saved was 5–12 times that lost by transpiration (Güerere *et al*. 1996). Similarly, in *T. calycinum*, 5–44 % of water was potentially conserved by CAM-cycling (Martin *et al*. 1988).

Leaf water balance in *E. milii* seems to rest on both recycling of respiratory CO_2_ and strict stomatal control, rather than on water supply from the succulent stem, as leaf FM/A remained unchanged after 16 days of drought and stem water content did not vary significantly during this time.

## Conclusions

In view of the observations presented here, *E. milii* can be considered as a CAM-cycling species that in watered plants shows diurnal, but not nocturnal, CO_2_ uptake and low ΔH^+^; plants under drought have very low *P*_N_, equally low ΔH^+^ and no net dark CO_2_ exchange. The significance of the operation of such a low CAM in *E. milii* resides in water conservation, rather than carbon acquisition. The occurrence of C_2_ photosynthesis remains to be demonstrated.

## Sources of Funding

Experiments were done with equipment acquired through grant PG-03.00.6524.2006 and technical assistance provided by grant PG 03.7381.2011-1 (CDCH-UCV).

## Conflicts of Interest Statement

None declared.
